# Enhancing Tetrahydrocannabinol’s Therapeutic Efficacy in Inflammatory Bowel Disease: The Roles of Cannabidiol and the Cannabinoid 1 Receptor Allosteric Modulator ZCZ011

**DOI:** 10.3390/ph18020148

**Published:** 2025-01-23

**Authors:** Dinesh Thapa, Mohan Patil, Leon N Warne, Rodrigo Carlessi, Marco Falasca

**Affiliations:** 1 Curtin Medical Research Institute, Curtin University, Perth, WA 6102, Australia; mohan.patil@postgrad.curtin.edu.au (M.P.); leon.warne@anaesthesia.vet (L.N.W.); rodrigo.carlessi@curtin.edu.au (R.C.); 2 College of Science, Health, Engineering and Education, Murdoch University, Perth, WA 6150, Australia; 3 Harry Perkins Institute of Medical Research, QEII Medical Centre and Centre for Medical Research, The University of Western Australia, Nedlands, WA 6009, Australia; 4 Department of Medicine and Surgery, University of Parma, 43125 Parma, Italy

**Keywords:** inflammatory bowel disease, DSS-induced ulcerative colitis, cannabinoid 1 receptor (CB1R), tetrahydrocannabinol (THC), CB1R allosteric modulator, ZCZ011, cannabidiol (CBD), glucagon-like peptide 1 (GLP-1), ammonia

## Abstract

**Background/Objectives:** Current inflammatory bowel disease (IBD) treatments focus on symptomatic relief, highlighting the need for innovative approaches. Dysregulation of the cannabinoid 1 (CB1) receptor, part of the endocannabinoid system, is linked to colitis. While tetrahydrocannabinol (THC) alleviates colitis via CB1 activation, its psychotropic effects limit clinical use. ZCZ011, a CB1R allosteric modulator, and cannabidiol (CBD), a non-psychoactive cannabinoid, offer alternatives. This study investigated combining sub-therapeutic THC doses with ZCZ011 or CBD in a murine model of dextran sodium sulphate (DSS)-induced colitis. **Methods:** Acute colitis was induced with 4% DSS for 7 days, followed by 3 days of water. Chronic colitis was modelled over 24 days with alternating DSS concentrations. The combination of 2.5 mg/kg THC with 20 mg/kg ZCZ011 or 10 mg/kg CBD was evaluated. Key markers were assessed to determine efficacy and safety, including disease activity index (DAI), inflammation, cytokine levels, GLP-1, and organ health. **Results:** DSS-induced colitis resulted in increased DAI scores, cytokines, organ inflammation and dysregulation of GLP-1 and ammonia. THC at 10 mg/kg significantly improved colitis markers but was ineffective at 2.5 and 5 mg/kg. ZCZ011 alone showed transient effects. However, combining 2.5 mg/kg THC with either 20 mg/kg ZCZ011 or 10 mg/kg CBD significantly alleviated colitis markers, restored colon integrity and reestablished GLP-1 homeostasis. This combination also maintained favourable haematological and biochemical profiles, including a notable reduction in colitis-induced elevated ammonia levels. **Conclusions:** This study demonstrates the synergistic potential of low-dose THC combined with CBD or ZCZ011 as a novel, effective and safer therapeutic strategy for ulcerative colitis.

## 1. Introduction

Ulcerative colitis (UC) is a chronic inflammatory bowel disease (IBD) characterized by recurring inflammation of the colonic mucosa, leading to symptoms such as abdominal pain, diarrhoea, rectal bleeding and weight loss [1]. Despite advances in therapeutic approaches, current treatments for UC are often associated with significant side effects and limited efficacy, highlighting the need for novel strategies [2]. The endocannabinoid system (ECS), which regulates various physiological functions, including gastrointestinal homeostasis and immune responses, has emerged as a promising therapeutic target for UC [3,4].

The ECS is an endogenous lipid signalling system that consists of G-protein-coupled receptors (GPCRs) including cannabinoid receptors (CB1 and CB2), endogenous ligands (endocannabinoids) and enzymes responsible for their synthesis and degradation [5]. CB1 receptors, primarily expressed in the central nervous system and also present in the gastrointestinal tract, play a role in modulating intestinal motility and inflammation [6]. In contrast, CB2 receptors are predominantly expressed in immune cells, where they regulate immune responses and inflammation [7,8]. Dysregulation of the ECS, including elevated expression of cannabinoid receptors and increased levels of the endocannabinoids anandamide (AEA) and 2-arachidonoylglycerol (2-AG), has been observed in UC patients, suggesting a potential compensatory mechanism to reduce inflammation [9,10].

Tetrahydrocannabinol (THC), the primary psychoactive component of *Cannabis sativa*, has been shown to alleviate pain, inflammation, cancers, etc., by acting as a partial agonist at both CB1 and CB2 receptors [11,12,13]. Preclinical studies have shown that THC can alleviate inflammation and promote tissue healing in models of colitis [14]. However, its therapeutic application is often limited by behavioural side effects such as anxiety, tolerance and cognitive impairment, which restrict its clinical utility [15,16,17]). Clinical trials on THC for inflammatory bowel disease (IBD), including Crohn’s disease and ulcerative colitis (UC), have shown mixed results. While some studies report significant improvements in quality of life and clinical scores, others found no notable impact on inflammatory markers compared to placebo [18]. Interestingly, low-dose THC in combination with other agents such as CB1 receptor allosteric modulators or cannabidiol (CBD), a non-psychoactive constituent of the cannabis plant, has shown promise in animal models of pain and inflammation [14,19], potentially offering benefits in terms of reduction of inflammation in UC as well. Allosteric modulators of CB1 receptors such as ZCZ011 are emerging as a novel approach to potentiate the efficacy of low-dose CB1 receptor orthosteric agonists such as THC or CP55,940 [20]. These modulators bind to allosteric sites on CB1 receptors, modulating receptor activity without directly activating the orthosteric site, thereby minimising the behavioural side effects associated with orthosteric activation of CB1 receptor [21,22]. This mechanism allows for the use of lower doses of THC in combination with the CB1 receptor allosteric modulator, reducing its adverse psychoactive effects while maintaining its anti-inflammatory benefits.

On the other hand, CBD also holds promise as a treatment for UC [23]. CBD has been shown to produce anti-inflammatory, antioxidant and immunomodulatory properties in different preclinical models [24,25]. Our previous study demonstrated the positive effects of CBD in managing ulcerative colitis [23]. However, a therapeutic dose of CBD interacts with the ECS in a complex manner targeting multiple receptors, including CB1, CB2, 5HT1A and GPR55 receptors, which may result in off-target side effects [25].

In this study, we investigated the potential of ZCZ011, a CB1 receptor allosteric modulator, and CBD to enhance the therapeutic efficacy of THC in a mouse model of dextran sodium sulphate (DSS)-induced colitis. The DSS model of colitis is a well-established experimental model for studying IBD pathophysiology such as abdominal pain, diarrhoea, colon injury, bleeding and body weight loss [26]. Moreover, this model was used to investigate the role of glucagon-like peptide 1 (GLP-1), a versatile biomarker of gut endocrine function, in the modulation of colon inflammation and glucose homeostasis in colitis [23]. In addition to its established roles in regulating glucose metabolism and body weight, GLP-1 has been shown to suppress pro-inflammatory cytokine production and promote gut barrier integrity [27,28]. These functions are particularly critical in managing colitis, where inflammation and compromised epithelial barriers contribute significantly to disease progression. Taking this into consideration, we hypothesize that combining a sub-therapeutic dose of THC with ZCZ011 or CBD produces anti-inflammatory effects, modulates GLP-1 levels locally and systemically, reduces colonic damage and improves treatment outcomes while minimizing the psychoactive side effects typically associated with a higher therapeutic dose of THC.

## 2. Results

### 2.1. Low-Dose Combination of THC and ZCZ011 Reduces Clinical Markers of Acute Colitis in a Mouse Model

**Figure 1:**
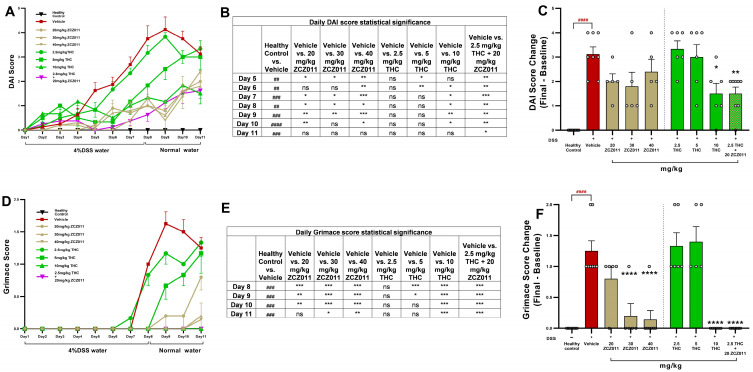
Effects of ZCZ011, THC and their combination on DAI and grimace scores in a DSS-induced acute colitis model. (**A**) Daily progression of the disease activity index (DAI) score in DSS-challenged mice treated with vehicle, ZCZ011 (20, 30 or 40 mg/kg), THC (2.5, 5 or 10 mg/kg) or a combination of 2.5 mg/kg THC with 20 mg/kg ZCZ011. (**B**) Statistical significance of daily DAI score. (**C**) Change in DAI score (final score minus baseline) at the study endpoint (day 11). (**D**) Daily progression of grimace scores reflecting pain behaviours. (**E**) Statistical significance of daily grimace score. (**F**) Change in grimace scores (final score minus baseline) at the study endpoint. Data are presented as mean ± SEM. #### *p* < 0.0001, ### *p* < 0.001, ## *p* < 0.01 vs. healthy control; **** *p* < 0.0001, *** *p* < 0.001 ** *p* < 0.01, * *p* < 0.05 vs. DSS vehicle group. ns—not statistically significant.

The therapeutic potential of cannabinoid-based treatments in colitis management has garnered significant attention. Here, we evaluate the efficacy of a low-dose combination of THC and ZCZ011 in reducing clinical and behavioural markers of acute colitis in a mouse model. Treatment with 4% DSS significantly increased the DAI score in the vehicle-treated group compared to healthy controls, indicating the development of severe colitis (Figure 1A,B; *p* < 0.0001). Monotherapy with ZCZ011 at 20, 30 or 40 mg/kg showed partial reductions in DAI score, diminishing effects after day 8 (Figure 1A,B). Similarly, low-dose THC (2.5 or 5 mg/kg) showed limited efficacy. However, high-dose THC (10 mg/kg) significantly reduced DAI scores compared to the vehicle group (*p* < 0.05). Notably, the combination of 2.5 mg/kg THC and 20 mg/kg ZCZ011 demonstrated a synergistic effect, resulting in a marked reduction in DAI score relative to the vehicle group (Figure 1B; *p* < 0.01) and comparable to the efficacy of high-dose THC without the need for higher dosing.

DSS treatment significantly elevated the grimace score in vehicle-treated mice, reflecting severe pain behaviours (Figure 1C,D; *p* < 0.0001). There was no significant reduction in grimace score with 20 mg/kg ZCZ011 or 2.5 and 5 mg/kg THC. In contrast, high-dose THC (10 mg/kg), 30 and 40 mg/kg ZCZ011 and the combination of 2.5 mg/kg THC with 20 mg/kg ZCZ011 significantly reduced grimace scores to near-baseline levels (Figure 1D; *p* < 0.0001). The combination therapy was particularly effective, achieving comparable pain relief to high-dose THC.

### 2.2. Lower-Dose Combination Treatments of THC with ZCZ011 or CBD Alleviated Clinical Colitis Scores in a Mouse Model of Chronic Colitis

**Figure 2:**
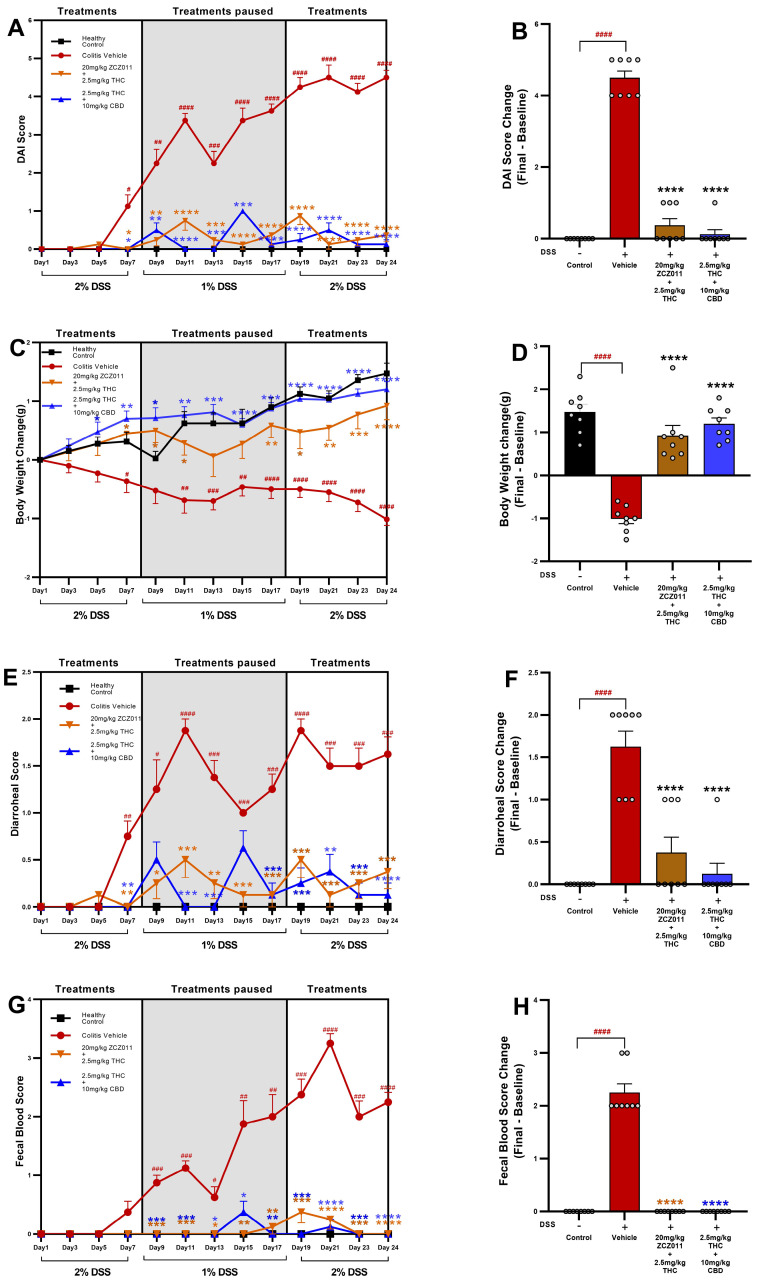
Therapeutic effects of THC combined with ZCZ011 or CBD on DAI scores in chronic colitis. The figure illustrates the progression and modulation of DSS-induced colitis across different treatment groups. (**A**,**B**) Disease activity index (DAI) scores. (**C**,**D**) Body weight loss. (**E**,**F**) Diarrhoea scores. (**G**,**H**) Faecal blood scores. Data presented as mean ± SEM, with significant differences denoted as: # *p* < 0.05, ## *p* < 0.01, ### *p* < 0.001, #### *p* < 0.0001 compared to the healthy control; and * *p* < 0.05, ** *p* < 0.01, *** *p* < 0.001, **** *p* < 0.0001 compared to the vehicle group.

The DAI scores were significantly higher in DSS-treated mice receiving the vehicle compared to the healthy control group, beginning on day 7 (Figure 2A,B; *p* < 0.0001). Treatment with 2.5 mg/kg THC in combination with 20 mg/kg ZCZ011 or 10 mg/kg CBD significantly attenuated DAI scores compared to the vehicle-treated group. By the end of the study, the change in DAI score from baseline was markedly reduced in both treatment groups compared to the vehicle (Figure 2B, *p* < 0.0001).

Vehicle-treated DSS mice exhibited significant body weight loss compared to the healthy control group from day 11 until the end of the study (Figure 2C,D; *p* < 0.0001). Treatment with 2.5 mg/kg THC in combination with 20 mg/kg ZCZ011 or 10 mg/kg CBD mitigated body weight loss, with treated groups demonstrating significant improvement in weight retention compared to the vehicle-treated group. Final body weight loss was significantly higher in the vehicle group compared to the control group (Figure 2D, *p* < 0.0001). Both treatment regimens (2.5 mg/kg THC + 20 mg/kg ZCZ011 or 2.5 mg/kg THC + 10 mg/kg CBD) maintained body weight at levels comparable to the healthy control group.

The vehicle-treated DSS group showed a significant increase in diarrhoeal score compared to the healthy control group (Figure 2E, *p* < 0.0001). Treatments with 2.5 mg/kg THC in combination with 20 mg/kg ZCZ011 or 10 mg/kg CBD markedly reduced diarrhoeal scores, demonstrating significant improvements compared to the vehicle-treated group (Figure 2F, *p* < 0.0001).

The faecal blood score, a key marker of colonic inflammation, was significantly higher in DSS vehicle-treated mice compared to the healthy control group (Figure 2G, *p* < 0.0001). Both treatment groups showed a substantial reduction in faecal blood scores compared to the vehicle group, with minimal or no observable bleeding by the end of the experiment (Figure 2H, *p* < 0.0001).

Overall, treatments with 2.5 mg/kg THC in combination with 20 mg/kg ZCZ011 or 10 mg/kg CBD demonstrated protective effects against DSS-induced colitis. These therapeutic interventions significantly improved clinical parameters, including DAI, body weight, diarrhoeal score and faecal blood score compared to the vehicle group.

### 2.3. A Lower-Dose Combination of THC and ZCZ011 Reduced Colon and Spleen Inflammation in Acute Colitis

**Figure 3:**
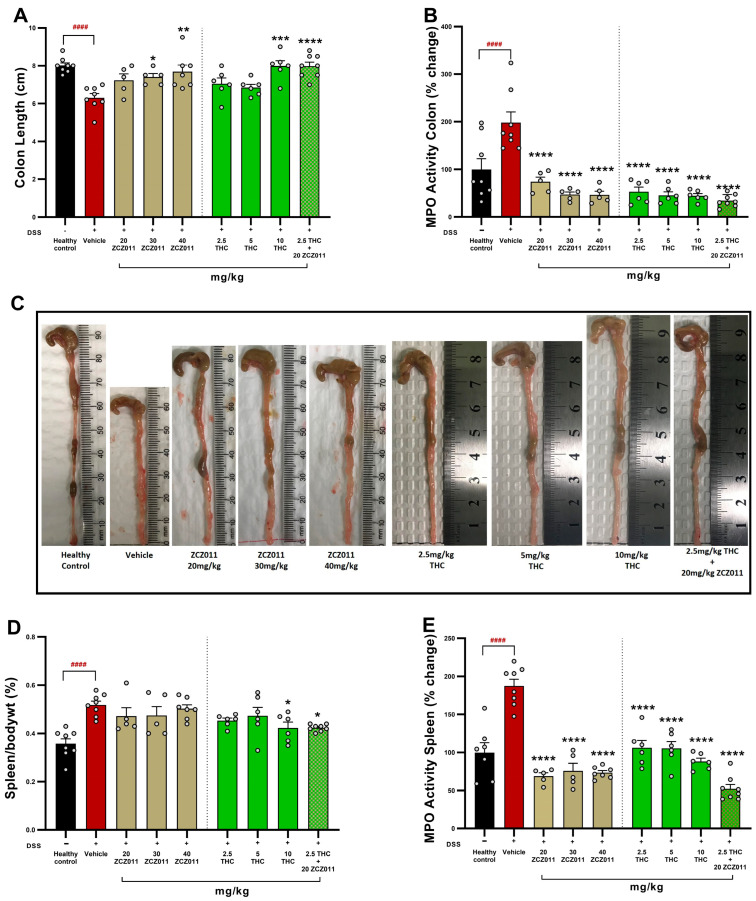
Effects of ZCZ011, THC and their combination on colon length, MPO activity and spleen weight in DSS-induced acute colitis. (**A**) Colon length measured at the study endpoint. (**B**) Percentage change in colon MPO activity. (**C**) Representative images of colons from each treatment group. (**D**) Spleen-to-body weight ratio as an indicator of systemic inflammation. (**E**) Percentage change in MPO activity in the spleen. Data are presented as mean ± SEM. #### *p* < 0.0001 vs. healthy controls; **** *p* < 0.0001, *** *p* < 0.001, ** *p* < 0.01, * *p* < 0.05 vs. DSS vehicle group.

Treatment with ZCZ011, THC and their combination significantly attenuated markers of colonic and splenic inflammation in DSS-induced acute colitis. DSS administration caused marked colonic shortening compared to healthy controls (Figure 3A; *p* < 0.0001). THC (5 and 10 mg/kg) and ZCZ011 (30 and 40 mg/kg) dose-dependently improved colon length, with the greatest improvement observed in the combination of 2.5 mg/kg THC with 20 mg/kg ZCZ011 (*p* < 0.0001 vs. vehicle). Myeloperoxidase (MPO) activity in the colon, a marker of neutrophil infiltration, was significantly elevated in the DSS vehicle group but normalised with all doses of THC and ZCZ011 (Figure 3B; *p* < 0.0001 vs. vehicle). Notably, the combination of 2.5 mg/kg THC with 20 mg/kg ZCZ011 synergistically reduced MPO activity to levels comparable to healthy controls.

The spleen-to-body weight percentage, an indicator of systemic inflammation, was significantly elevated in the DSS vehicle group (Figure 3D; *p* < 0.0001). While individual treatments provided modest improvements, the THC-ZCZ011 combination restored spleen weight to near-normal levels (*p* < 0.05). MPO activity in the spleen mirrored these results with significant reductions across all treatment groups (Figure 3E; *p* < 0.0001) and the THC-ZCZ011 combination demonstrating the most pronounced effect.

This finding demonstrates that the combination of low-dose THC (2.5 mg/kg) with ZCZ011 (20 mg/kg) effectively ameliorates colonic and systemic inflammation, offering potential advantages over individual treatments.

### 2.4. THC Combination with Either ZCZ011 or CBD Reduced Colonic and Splenic Inflammation in Chronic Colitis

**Figure 4:**
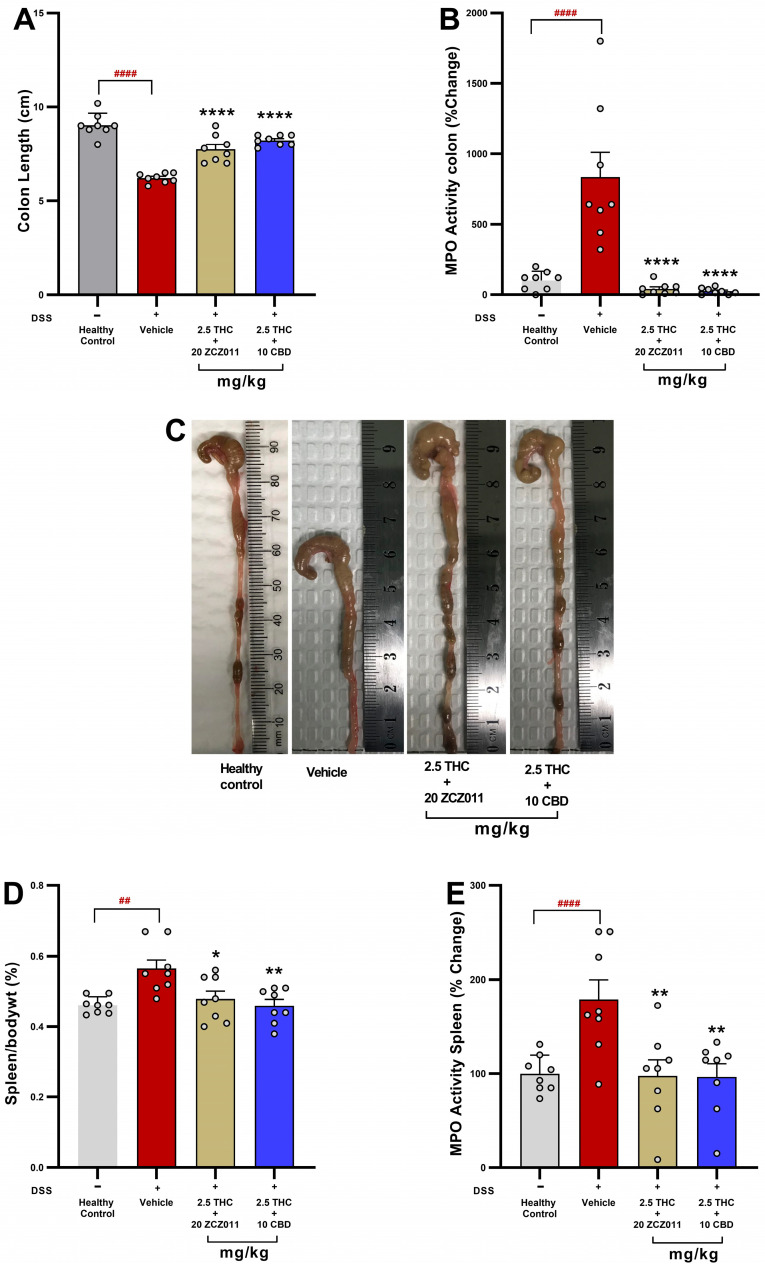
Effects of combination therapies on markers of colitis severity and systemic inflammation. The figure illustrates the modulation of colon and spleen inflammation in chronic colitis across different treatment groups. (**A**) Colon length. (**B**) Colonic MPO activity. (**C**) Representative images of colons. (**D**) Spleen-to-body weight percentage. (**E**) Splenic MPO activity. Data expressed as mean ± SEM, with significant differences denoted as ## *p* < 0.01, #### *p* < 0.0001 compared to the healthy control; and * *p* < 0.05, ** *p* < 0.01, **** *p* < 0.0001 compared to the vehicle group.

To evaluate the therapeutic potential of combining sub-therapeutic doses of THC with CBD or ZCZ011, key markers of colitis severity and systemic inflammation were assessed. Treatment with either 2.5 mg/kg THC + 20 mg/kg ZCZ011 or 2.5 mg/kg THC + 10 mg/kg CBD significantly increased colon length compared to the vehicle-treated DSS group (Figure 4A; *p* < 0.0001), which exhibited marked colon shortening relative to the healthy control group (*p* < 0.0001). Colonic MPO activity, which was significantly increased in the vehicle-treated group compared to the healthy control group (Figure 4B; *p* < 0.0001), was markedly suppressed by the combination treatments compared to the vehicle-treated DSS group (*p* < 0.0001), reducing activity to baseline levels. Representative images (Figure 4C) illustrate the improvement in colon morphology with the combination therapies compared to the vehicle -treated group. 

The combination therapies also reduced systemic inflammation, as indicated by decreased spleen-to-body weight percentage and splenic MPO activity. Spleen weight, which was significantly increased in the vehicle group compared to the healthy control group (Figure 4D; *p* < 0.01), was significantly reduced in both treatment groups compared to the vehicle-treated group (*p* < 0.05 and *p* < 0.01, respectively). Similarly, splenic MPO activity was significantly lower in the treatment groups compared to the vehicle-treated group (Figure 4E; *p* < 0.01).

### 2.5. A Sub-Therapeutic Dose Combination of THC with ZCZ011 or CBD Reduced Colonic Cytokine and Chemokine Levels in Chronic Colitis

To evaluate the anti-inflammatory effects of sub-therapeutic dose combinations of THC with ZCZ011 or CBD, levels of pro-inflammatory cytokines and chemokines in colonic tissue were assessed.

**Figure 5:**
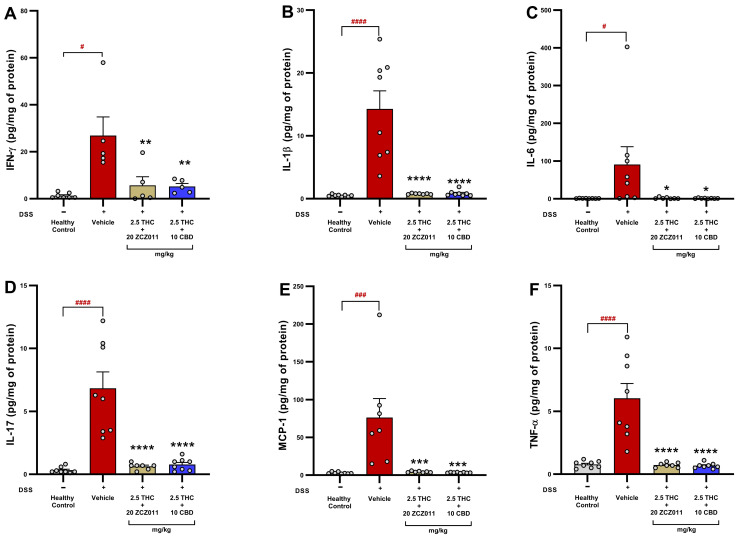
Effects of combination treatments on colonic inflammatory cytokine levels in DSS-induced chronic colitis**.** The figure presents the levels of pro-inflammatory cytokines and chemokines in colonic tissue across different treatment groups. (**A**) IFN-γ. (**B**) IL-1β. (**C**) IL-6. (**D**) IL-17. (**E**) MCP-1. (**F**) TNF-α. Data are presented as mean ± SEM. Statistical significance: # *p* < 0.05, ### *p* < 0.001, #### *p* < 0.0001 vs. healthy control; * *p* < 0.05, ** *p* < 0.01, *** *p* < 0.001, **** *p* < 0.0001 vs. DSS vehicle group.

Colonic IFN-γ levels were significantly elevated in the DSS + vehicle group compared to the healthy control group (Figure 5A; *p* < 0.05). Treatment with 2.5 mg/kg THC, either with 20 mg/kg ZCZ011 or 10 mg/kg CBD, significantly reduced IFN-γ levels compared to the vehicle-treated group (*p* < 0.05). Similarly, IL-1β levels were markedly increased in the DSS + vehicle group relative to healthy controls (Figure 5B; *p* < 0.0001). Both combination treatments significantly suppressed IL-1β levels to near-baseline levels compared to the vehicle-treated group (*p* < 0.0001). Vehicle-treated DSS mice displayed a substantial increase in IL-6 levels compared to healthy controls (Figure 5C; *p* < 0.05). Both combination treatments significantly reduced IL-6 levels relative to the vehicle group (*p* < 0.05). The DSS + vehicle group exhibited a pronounced elevation in IL-17 levels compared to the healthy control group (Figure 5D; *p* < 0.0001). Both 2.5 mg/kg THC + ZCZ011 and 2.5 mg/kg THC + CBD treatments resulted in a significant reduction in IL-17 levels (*p* < 0.0001). MCP-1 levels were significantly increased in the DSS + vehicle group compared to the healthy control group (Figure 5E; *p* < 0.001). Both THC-based combination treatments significantly decreased MCP-1 levels relative to the vehicle group (*p* < 0.001). DSS + vehicle mice showed a marked increase in TNF-α levels relative to the healthy controls (Figure 5F; *p* < 0.0001). Treatment with THC in combination with ZCZ011 or CBD significantly suppressed TNF-α levels compared to the vehicle-treated group (*p* < 0.0001).

These findings indicate that sub-therapeutic dose combinations of THC with ZCZ011 or CBD effectively suppress the production of key inflammatory mediators, highlighting their potential as anti-inflammatory therapies for chronic colitis.

### 2.6. Lower-Dose THC and ZCZ011 Combination Restores GLP-1 Levels, Mitigating Weight Loss and Blood Sugar Dysregulation in Acute Colitis

**Figure 6:**
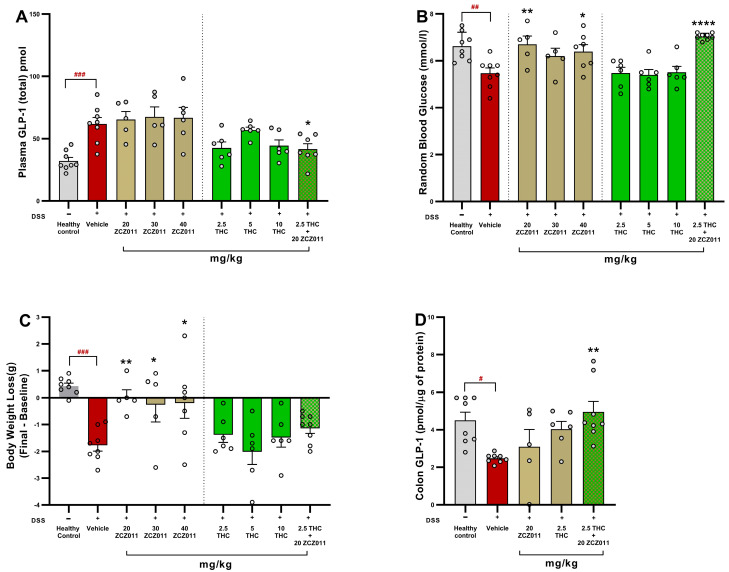
Effects of THC, ZCZ011 and their combination on plasma GLP-1 levels, body weight, blood glucose and colonic GLP-1 in DSS-induced acute colitis. (**A**) Plasma GLP-1 (total) levels. (**B**) Random blood glucose levels. (**C**) Change in body weight. (**D**) Colonic GLP-1 levels. Data are presented as mean ± SEM. Statistical significance: # *p* < 0.05, ## *p* < 0.01 and ### *p* < 0.001 compared to healthy controls and * *p* < 0.05, ** *p* < 0.01, **** *p* < 0.0001 compared to DSS vehicle group.

GLP-1, a hormone involved in glucose homeostasis and appetite regulation, is also critical in modulating inflammation and maintaining gut integrity during colitis [28,29]. Here, we investigate the effects of a low-dose combination of THC and ZCZ011 on GLP-1 levels, body weight and blood glucose regulation in a DSS-induced acute colitis model. Plasma GLP-1 levels were significantly increased in the DSS vehicle group compared to healthy controls (Figure 6A; *p* < 0.001). While monotherapies with ZCZ011 (20, 30 or 40 mg/kg) or THC (2.5, 5 or 10 mg/kg) did not significantly restore plasma GLP-1 levels, the combination of 2.5 mg/kg THC with 20 mg/kg ZCZ011 significantly restored plasma GLP-1 levels to near-healthy control levels (*p* < 0.05 vs. vehicle).

DSS treatment caused a significant reduction in random blood glucose levels in the vehicle group compared to healthy controls (Figure 6B; *p* < 0.01). Treatments with 20 or 40 mg/kg ZCZ011 normalised these levels (*p* < 0.01, *p* < 0.05, respectively. While monotherapy with THC had no significant improvement in glucose levels, the combination treatment with 2.5 mg/kg THC and 20 mg/kg ZCZ011 significantly normalised blood glucose levels (*p* < 0.0001).

Body weight loss in the vehicle-treated DSS group was significantly higher compared to the healthy control group (Figure 6C; *p* < 0.001). Treatment with ZCZ011 at doses of 20, 30 or 40 mg/kg significantly mitigated weight loss, with *p*-values of <0.01, <0.05 and <0.05, respectively. The combination of 2.5 mg/kg THC with 20 mg/kg ZCZ011 demonstrated a moderate effect in the mitigation of body weight loss.

In addition to plasmatic GLP-1 levels, we also measured colonic GLP-1 levels. GLP-1 was reduced significantly in vehicle-treated DSS mice compared to healthy controls (Figure 6D; *p* < 0.05). While 2.5 mg/kg THC or 20 mg/kg ZCZ011 had no significance on their own, their combination treatment significantly restored colonic GLP-1 levels (*p* < 0.01).

These results suggest that the combination of sub-therapeutic THC and ZCZ011 exhibits synergistic effects in restoring GLP-1 levels and mitigating colitis-associated body weight loss and blood glucose dysregulation.

### 2.7. The Combination Treatments of THC with ZCZ011 or CBD Normalise GLP-1 Levels and Maintain Glucose Homeostasis in Chronic Colitis

**Figure 7:**
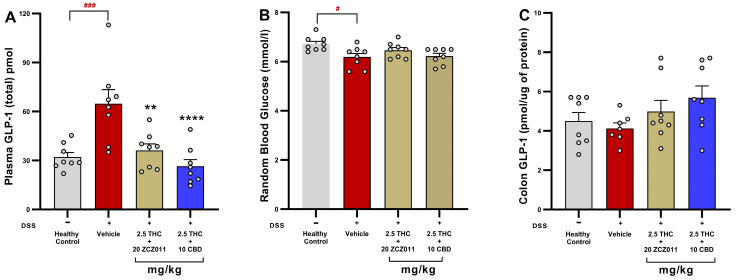
Effects of combination therapies on GLP-1 levels and glucose regulation in DSS-induced chronic colitis. (**A**) Plasma GLP-1 levels. (**B**) Random blood glucose levels. (**C**) Colonic GLP-1 levels (pmol/µg of protein). Data are presented as mean ± SEM with statistical significance # *p* < 0.05 and ### *p* **<** 0.001 compared to health control groups and ** *p* < 0.01, ****p < 0.0001 compared to the vehicle-treated DSS group.

To determine the impact of cannabinoid-based therapies on GLP-1 levels and glucose regulation, plasma and colonic GLP-1 levels, as well as random blood glucose levels, were analysed. Chronic colitis significantly elevated plasma GLP-1 levels in the vehicle-treated group compared to healthy controls (Figure 7A; *p* < 0.001). Treatment with 2.5 mg/kg THC + 20 mg/kg ZCZ011 or 2.5 mg/kg THC + 10 mg/kg CBD effectively normalised plasma GLP-1 levels compared to the vehicle group (*p* < 0.01 and *p* < 0.0001, respectively).

Random blood glucose levels were significantly reduced in the vehicle-treated DSS group compared to healthy controls (Figure 7B; *p* < 0.05). However, both combination treatments increased glucose levels comparable to healthy controls, indicating a protective effect on glucose homeostasis.

In the colon, GLP-1 levels remained stable across all groups, with no significant differences observed (Figure 7C), suggesting that the therapies specifically targeted systemic GLP-1 dysregulation rather than local colonic GLP-1 production.

### 2.8. Impact of THC and ZCZ011 Alone or in Combination in Haematology, Liver and Kidney Function Parameters in Acute Colitis

No significant changes were observed in haematological parameters across the groups, except for a mild decrease in platelet and procalcitonin levels in the vehicle-treated DSS group compared to the healthy control. These abnormalities were restored to levels comparable to the healthy control with the combination treatment of 2.5 mg/kg THC and 20 mg/kg ZCZ011 (Table 1; *p* < 0.01 compared to vehicle).

**Figure 8:**
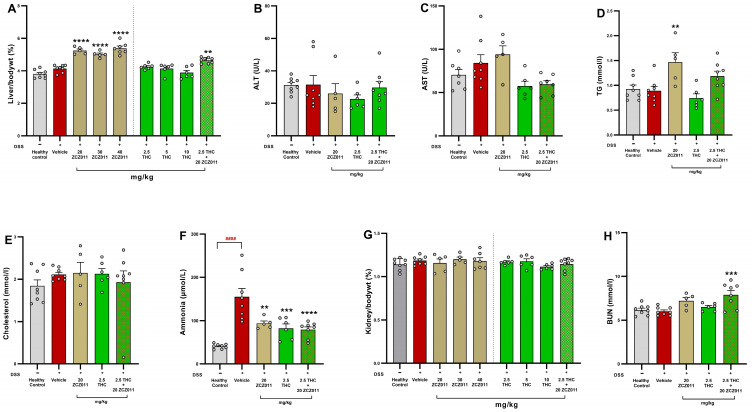
Effects of ZCZ011 and THC alone or in combination on liver and kidney function parameters. The figure illustrates the effects of treatments in liver and kidney function parameters. (**A**) Liver-to-body weight ratio (%). (**B**) Plasma ALT levels (U/L). (**C**) Plasma AST levels (U/L). (**D**) Plasma TG levels (mmol/L). (**E**) Plasma cholesterol levels (mmol/L). (**F**) Plasma ammonia levels (μmol/L). (**G**) Kidney-to-body weight ratio (%). (**H**) Plasma BUN levels (mmol/L). Data are presented as mean ± SEM. Statistical significance was determined using one-way ANOVA followed by Dunnet post hoc test. #### *p* < 0.0001 versus healthy control; ** *p* < 0.01, *** *p* < 0.001, **** *p* < 0.0001 versus vehicle.

The percentage of liver-to-body weight ratio significantly increased in DSS-treated mice administered ZCZ011 at 20, 30 and 40 mg/kg compared to the vehicle group (Figure 8A; *p* < 0.0001). Similarly, a modest but significant increase was observed in the group receiving a combination of 2.5 mg/kg THC and 20 mg/kg ZCZ011 (*p* < 0.01). Plasma levels of ALT and AST showed no significant differences among treatment groups, indicating the absence of hepatotoxicity under these experimental conditions (Figure 8B,C, respectively). Triglyceride (TG) levels were significantly elevated in the group treated with 20 mg/kg ZCZ011 (Figure 8D; *p* < 0.01) compared to the vehicle group while no significant changes were observed in cholesterol levels across all experimental groups (Figure 8E). The vehicle-treated DSS group had a substantial increase in plasma ammonia levels compared to the healthy control group (Figure 8F; *p* < 0.0001). This increase was notably reduced in the 20 mg/kg ZCZ011 group (*p* < 0.01), the 2.5 mg/kg THC group (*p* < 0.001) and the combination group treated with 2.5 mg/kg THC and 20 mg/kg ZCZ011 (*p* < 0.0001). The percentage of kidney-to-body weight ratios remained stable across all groups, suggesting no nephrotoxicity (Figure 8G). However, plasma BUN levels were significantly elevated in the combination group receiving 2.5 mg/kg THC and 20 mg/kg ZCZ011 (Figure 8H; *p* < 0.001).

### 2.9. A Sub-Therapeutic Dose Combination of THC with ZCZ011 or CBD Offers a Desirable Safety Profile

The combination treatments of THC with ZCZ011 or CBD did not induce haematological abnormalities in chronic colitis (Table 2)

**Figure 9:**
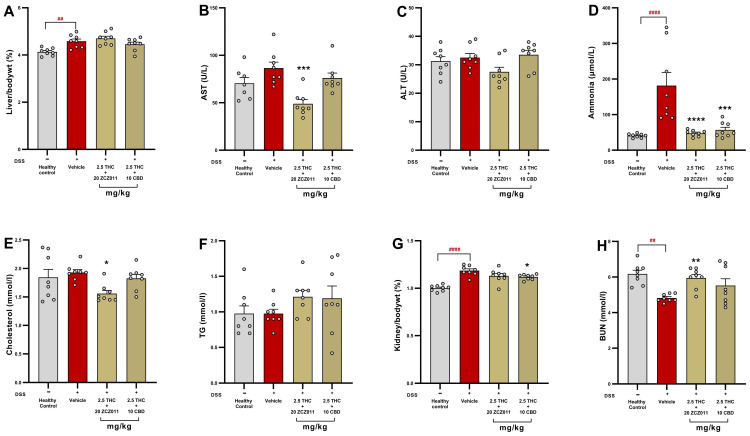
Effects of THC combined with ZCZ011 or CBD on hepatic and renal parameters in DSS-induced chronic colitis. (**A**) Liver-to-body weight (%). (**B**) Plasma AST levels (U/L). (**C**) Plasma ALT levels (U/L). (**D**) Plasma ammonia levels (µmol/L). (**E**) Plasma cholesterol levels (mmol/L). (**F**) Plasma triglyceride levels (mmol/L). (**G**) Kidney-to-body weight ratio (%) and (**H**) Plasma blood urea nitrogen (BUN) levels (mmol/L). Data are presented as mean ± SEM. Statistical significance: ## *p* < 0.01, #### *p* < 0.0001 vs. healthy control; * *p* < 0.05, ** *p* < 0.01, *** *p* < 0.001, **** *p* < 0.0001 vs. vehicle-treated DSS group.

The effects of THC combined with ZCZ011 or CBD on hepatic and renal parameters in DSS-induced chronic colitis were assessed. Vehicle-treated DSS mice significantly increased liver-to-body weight ratio compared to healthy control (Figure 9A; *p* < 0.01). While there was no statistical difference in levels of AST (Figure 9B) and ALT (Figure 9C), plasma ammonia levels (Figure 9D) were significantly elevated in the vehicle-treated group compared to healthy controls (*p* < 0.0001). AST levels were significantly reduced by THC + ZCZ011 compared to the vehicle group (*p* < 0.001) whereas ALT levels remained unaffected across all groups. Both combination treatments, 2.5 mg/kg + 20 mg/kg ZCZ011 and 2.5 mg/kg THC + 10 mg/kg CBD, significantly reduced plasma ammonia levels compared to the vehicle-treated DSS group (*p* < 0.0001 and *p* < 0.001, respectively).

There was no statistical difference in plasma cholesterol (Figure 9E) and triglyceride (TG) levels (Figure 9F) in the vehicle-treated DSS group compared to the healthy control. Treatment with THC + ZCZ011 significantly reduced cholesterol levels, while both combinations did not affect TG levels.

Chronic DSS exposure also caused significant renal hypertrophy, as evidenced by an increased kidney-to-body weight percentage in the vehicle group compared to healthy control (Figure 9G; *p* < 0.0001). BUN levels were significantly reduced in the chronic vehicle group compared to healthy control (Figure 9H; *p* < 0.01). THC combination therapies attenuated kidney hypertrophy and BUN levels compared to the vehicle-treated group, indicating renal protection.

## 3. Discussion

Inflammatory bowel disease (IBD) remains challenging to manage due to the limitations of current therapies, including biologics and immunosuppressive agents, which often provide incomplete remission, cause adverse effects and have limited accessibility due to high cost [30,31]. Cannabis and cannabinoids have been utilized globally as alternative therapies for managing gastrointestinal symptoms such as diarrhoea, pain and inflammation [18]. This study highlights a novel therapeutic approach targeting the endocannabinoid system (ECS)—an endogenous lipid signalling network that regulates pain, inflammation and metabolism [5]—using sub-therapeutic doses of THC combined with ZCZ011 or CBD. By leveraging the ECS’s ability to modulate immune responses, this strategy overcomes the psychoactive concerns associated with high-dose THC while delivering potent anti-inflammatory and metabolic benefits [20].

Using well-established DSS-induced acute and chronic colitis models, our study demonstrates that low-dose THC (2.5 mg/kg) in combination with ZCZ011 (20 mg/kg) or CBD (10 mg/kg) significantly attenuates disease severity, restores tissue integrity and improves systemic markers of inflammation (Figure 1 and Figure 2). In both colitis models, the combination treatments effectively reduced disease activity index (DAI), body weight loss, diarrhoea severity and faecal blood, achieving comparable efficacy to a therapeutic dose of THC (10 mg/kg). This is in line with the previous findings where the modulation of ECS by CBD or HU308, a CB2 receptor agonist, significantly reduced colitis in DSS-induced colitis [23]. Similarly, the combination of THC and CBD has been shown to reduce colitis in a rat model of 2,4,6-trinitrobenzene sulphonic acid (TNBS)-induced colitis in rats, suggesting that the effects observed in our study are not model-specific [14]. These findings align with earlier evidence on cannabinoid-based therapies, reinforcing the ECS as a viable therapeutic target in IBD [32,33]. Importantly, our results highlight the consistency of these therapeutic effects across acute and chronic disease states, further supporting their translational relevance.

After observing significant improvements in clinical markers, we examined the health of the colon and spleen, as both organs are known to be inflamed in DSS-induced colitis [23,34]. Combination treatments effectively mitigated colonic shortening, reduced neutrophil infiltration (measured via MPO activity) and normalised spleen weight in both acute and chronic colitis models (Figure 3 and Figure 4). This dual effect on local and systemic inflammation highlights the anti-inflammatory synergy of THC with ZCZ011 or CBD and suggests ECS modulation as a critical mechanism underlying these benefits. Consistent reductions in colon shortening, spleen inflammation and MPO activity across both models emphasize the potential of cannabinoid therapies to target multiple inflammatory pathways [23,35]. Targeting ECS was also shown to be beneficial in other models of pain and inflammatory conditions [19,32,36].

One of the novel insights of this study is the impact of combination therapies on pro-inflammatory cytokines and metabolic health. Pro-inflammatory cytokines, including IL-6, TNF-α, IL-1β, IL-17, IFN-γ and chemokine MCP-1, are key drivers of IBD pathology, contributing to immune cell recruitment, tissue damage, mucosal injury and systemic inflammation [37,38]. Our previous study demonstrated a significant elevation of IL-6, TNF-α, IL-1β, IL-17, IFN-γ and MCP-1 in colitis and showed that activation of the CB2 receptor by HU308 significantly reduces these cytokine levels [23]. Interestingly, combination treatments in this study significantly reduced these cytokines and chemokines (Figure 5), which correlated with improvements in clinical markers and tissue integrity. These results are consistent with the established role of cannabinoids in suppressing cytokine storms, primarily through CB2 receptor activation [23,39,40].

We further explored the effects of these therapies on GLP-1, an incretin hormone known for its anti-inflammatory and glucose-regulatory properties [28]. Colitis is often associated with metabolic disturbances, including impaired glucose homeostasis, body weight regulation and the development of non-alcoholic fatty liver disease (NAFLD) [41,42]. Elevated levels of cytokines have been also linked to abnormal glucose and lipid metabolism [43]. Beyond glucose regulation, GLP-1 has been also shown to reduce inflammation [27,28,44] and the expression of cytokines at local and systemic levels [45,46]. Recent findings also highlight the pivotal role of GLP-1 in maintaining metabolic health, which is often disrupted during colitis [47]. In our models, we observed increased plasma GLP-1 levels but decreased colonic levels, which is likely due to inflammation-induced dysregulation of enteroendocrine L-cells, the primary source of GLP-1 [48]. DSS-induced epithelial damage and inflammation may impair local GLP-1 synthesis or retention in the colon. Meanwhile, systemic inflammation and cytokine signalling (e.g., IL-6, TNF-α) could stimulate L-cells in unaffected gut regions to release GLP-1 into the bloodstream as a compensatory response [49]. Additionally, GLP-1’s systemic increase may serve as a protective mechanism to maintain gut integrity and modulate inflammation, despite its reduced colonic levels due to degradation by dipeptidyl peptidase-4 (DPP-4) or local retention issues. Interestingly, a key novel finding of this study is the normalization of both plasmatic and colonic GLP-1 levels following the combination treatments (Figure 6 and Figure 7). This restoration was accompanied by improvements in blood glucose homeostasis and body weight stability, indicating that these therapies not only ameliorate inflammation but also address colitis-associated metabolic dysregulation. This observation aligns with prior evidence indicating that chronic colonic inflammation can impair glucose utilization, leading to hypoglycaemia and metabolic stress [50]. Emerging evidence suggests that cannabinoids can modulate GLP-1 pathways to restore metabolic balance [51,52,53]**,** and our findings reinforce this dual therapeutic potential. We reported similar findings in our previous study, where the cannabinoids CBD and HU308 normalised both plasmatic and colonic GLP-1 levels [23]. Future studies should aim to elucidate the precise mechanisms underlying these effects, focusing on downstream signalling pathways in GLP-1 regulation and glucose metabolism, quantification of L-cells and (DPP-4) enzyme activity in the colon.

Consistent with the beneficial effects of the combination treatments in colitis markers, the safety profile of combination therapies was assessed through haematological, liver and kidney profiles. Acute colitis led to modest platelet and procalcitonin dysregulation, likely reflecting systemic inflammation, which was effectively restored to control levels by a combination of THC and ZCZ011 treatment (Table 1). No haematological dysregulation was observed in chronic colitis (Table 2). Liver biochemistry remained stable across all groups, with no hepatotoxicity observed with the treatments (Figure 8 and Figure 9), indicating no long-term toxicity. Additionally, the treatments significantly reduced plasma ammonia levels, which were elevated in vehicle-treated groups. Elevated ammonia levels are associated with various diseases, including neuropsychiatric disorders such as depression, anxiety and Alzheimer’s disease [54,55,56,57,58], as well as liver pathologies, which may manifest as extraintestinal complications of IBD [58,59,60]. By mitigating ammonia dysregulation, combination therapies may offer broader protective effects against IBD-associated systemic complications [60]. Cannabinoid therapies are increasingly recognized for their hepatoprotective [61] and renoprotective activity [62,63,64], primarily through their antioxidative and anti-inflammatory properties [65,66,67,68]. The DSS-induced colitis model is widely used for studying IBD; however, its relevance to human disease is limited by differences in aetiology, including genetic and environmental factors. Despite these limitations, the model effectively replicates key pathological features of IBD, such as clinical symptoms, epithelial barrier disruption, immune cell infiltration and pro-inflammatory cytokine production, making it a valuable tool for preclinical research. Furthermore, the chronic model employed in this study captures the phases of relapse and remission, closely resembling the clinical progression of human IBD. Nevertheless, incorporating complementary models or clinical studies to validate the findings of this study would enhance their translational relevance.

## 4. Materials and Methods

### 4.1. Animal Care and Use

All animal procedures followed the Australian Code for the Care and Use of Animals for Scientific Purposes (8th Edition, 2013, updated 2021) and were approved by the Curtin University Animal Research Ethics Committee (Approval No.: ARE2022-20). Female BALB/c mice (8–12 weeks old, ARC and Ozgene, Perth, Australia) were used after one week of acclimatization in specific pathogen-free (SPF) conditions. Mice were housed (5 per cage) under controlled temperature and humidity, with a 12-h light/dark cycle and ad libitum access to standard diet and water. Female mice were exclusively used in this study due to the consistent and reproducible nature of colitis induction in this sex [26]. The inclusion of male mice could have introduced confounding factors, as their higher aggression levels and tendency to fight may lead to increased stress. However, restricting the study to female mice limits the generalizability of the findings across sexes. To address this limitation, future studies will include both male and female mice to better understand potential sex-based differences in response to the treatments tested.

### 4.2. Dextran Sodium Sulphate (DSS) Solution Preparation

Colitis-grade Dextran Sulphate Sodium (MW 36,000–50,000 Da, Lot No. S8634; MP Biomedicals, Santa Ana, CA, USA) was prepared at 4% (*w*/*v*) concentration in autoclaved drinking water.

### 4.3. Induction of Colitis

Acute and chronic colitis were induced as per our previously published paper [23]. Briefly, for the induction of acute colitis, mice received 4% DSS solution as their sole drinking water for 7 days, followed by regular water for 3 days. Fresh DSS solution was provided every 3 days. Control mice received normal drinking water throughout. For chronic colitis, mice were administered 2% DSS for 7 days, 1% DSS for 10 days and 2% DSS for another 7 days. This regimen models the relapse and remission phases of human colitis. Control mice received water only.

### 4.4. Experimental Design and Pharmacological Treatments

**Figure 10:**
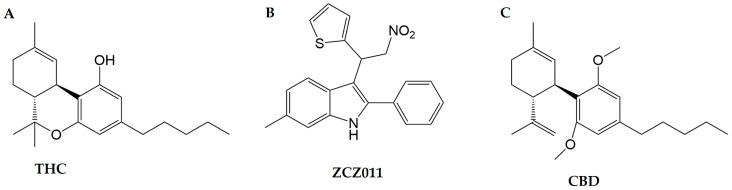
Chemical structure of THC (**A**), ZCZ011 (**B**) and cannabidiol (**C**).

Mice were randomly allocated into control, DSS vehicle and treatment groups. Treatments and data collection were blinded. For the acute colitis, mice were treated with delta-9 tetrahydrocannabinol (THC, Cayman Chemical, Ann Arbor, MI, USA; Figure 10A) at 2.5, 5 or 10 mg/kg or ZCZ011 (Axon Medchem, Groningen, the Netherlands; Figure 10B) at 20, 30 or 40 mg/kg or the combination of 2.5 mg/kg THC and 20 mg/kg ZCZ011 intraperitoneally (i.p.) once daily for 11 days. For chronic colitis, mice were treated with the combination of 2.5 mg/kg THC either with 20 mg/kg ZCZ011 or 10 mg/kg cannabidiol (CBD, Commonwealth Extracts, Louisville, KY, USA; Figure 10C) once daily during two 2% DSS phases (i.e., days 1–7 and days 18–24). Treatments were paused during the 1% DSS phase. The doses for THC, ZCZ011 and CBD were selected based on prior literature and our research in murine models of colitis, aiming to minimize the psychoactive side effects associated with higher doses of THC. THC was administered at doses up to 10 mg/kg, which have demonstrated anti-inflammatory efficacy without significant psychoactive effects [14,69]. ZCZ011 was given up to 40 mg/kg, as shown to be effective as a CB1 receptor allosteric modulator without psychoactive side effects [20], with an additional lower dose (20 and 30 mg/kg) tested in this study. The CBD dose (10 mg/kg) was selected based on our previous study [23]

### 4.5. Colitis Markers Assessment

#### 4.5.1. Disease Activity Index Score

DAI score was evaluated by summing the body weight loss, stool consistency/diarrhoea and rectal bleeding as described previously, including in our paper [23,26]. Table 3 outlines the criteria used to score DAI.

#### 4.5.2. Pain Behaviours

Pain was assessed using the grimace scoring method (GSM), focusing on facial expressions and body postures such as orbital tightening, nose bulge, whisker change, cheek bulge and ear position; changes in body position and movement were assessed as overall indicators of pain or discomfort as previously reported [70]. Table 4 outlines the criteria used to score pain-related behaviours in the mice.

### 4.6. Random Blood Glucose

Blood glucose level across the groups was measured on the final day (day 11 or day 24) using a handheld glucometer (Accu-Chek Performa^®^, Roche Ltd., Basel, Switzerland).

### 4.7. Euthanasia and Tissue Collection

On day 11 (acute) or day 24 (chronic), mice were anaesthetized, blood was collected via cardiac puncture, mice were euthanised by cervical dislocation, and organs (colon, spleen, liver, kidneys) were harvested for further analysis.

### 4.8. Measurement of Colon Length

Colon shortening, an indicator of inflammation, was measured from the cecum to the rectum. Briefly, following euthanasia, the entire colon, from the cecum to the rectum, was carefully removed and placed on a clean, flat surface. The length of the colon, from the cecal tip (proximal colon) to the rectum (distal colon), was measured using a ruler and images were captured. Colon lengths across all experimental groups were recorded under the same conditions for analysis. Following measurement, the colon was thoroughly washed with cold 1% Phosphate buffered solution (PBS) using a syringe fitted with an oral gavage needle and colon tissue was snap-frozen in liquid nitrogen and stored at −80 °C for further analysis.

### 4.9. Organ to Body Weight Ratio

Liver, spleen and kidney weights were measured and expressed as a percentage of body weight.

### 4.10. Myeloperoxidase (MPO) Activity

MPO levels in the colon and spleen, indicating neutrophil activity, were measured using a spectrophotometric assay as previously reported [23]. Briefly, colon and spleen samples were collected, blotted dry, weighed and placed in potassium phosphate buffer (pH 6.0) with 0.5% hexadecyltrimethylammonium bromide (HTAB; Sigma-Aldrich, St. Louis, MO, USA) at a ratio of 100 mL per 5 g of tissue. The tissues were manually crushed, homogenized and sonicated. After centrifuging the homogenate for 15 min at maximum speed (4 °C), the supernatant was collected. A 7 μL aliquot of the supernatant was added to 200 μL of 50 mM potassium phosphate buffer (pH 6.0) containing 0.167 mg/mL O-dianisidine hydrochloride (Sigma-Aldrich, St. Louis, MO, USA) and 0.5 μL of 1% H_2_O_2_. Myeloperoxidase (MPO) activity was measured at 460 nm by recording absorbance changes over 1 min using a multimode 96-well plate reader (PerkinElmer, Hongkong, China).

### 4.11. Cytokine and Chemokine Quantification

Pro-inflammatory and anti-inflammatory cytokines (TNFα, IL-1β, IL-6, IL-12, CCL2 and IL-10) in colon tissues were quantified using a multiplex assay kit (MCYTOMAG-70 K, Merck Millipore, Germany) as previously reported [23]. The manufacturer’s instructions were followed to carry out the assay. Briefly, frozen colon tissues (−80 °C) were homogenized in RIPA buffer (Sigma-Aldrich), sonicated and centrifuged at maximum speed for 15 min. The supernatant was collected, and protein concentration was determined using a Micro BCA assay (ThermoFisher Scientific, Waltham, MA, USA). Readings were obtained in MagPix Luminex100 reader (Luminex Corporation 12212 Technology Blvd. Austin, TX, USA). Values are expressed in pg/mg of protein.

### 4.12. GLP-1 Measurement

Plasma and colon GLP-1 levels were measured using a commercially available ELISA kit (EMD Millipore, Darmstadt, Germany). The assay was performed according to the manufacturer’s instructions and readings were obtained using a multimode 96-well plate reader. Results were expressed as picomolar (pmol) for plasma samples and pmol/µg of protein for colon lysates.

### 4.13. Haematology and Biochemistry

Fresh whole blood in heparin-coated tubes was analysed for haematological parameters using an automated blood analyser (BC-2800, Mindray, Shenzhen, China). Biochemical markers (ALT, AST, BUN, albumin, globulin, creatinine, ammonia and total protein) were measured in plasma using an automated biochemistry analyser (Element DC, Heska, Loveland, CO, USA).

### 4.14. Statistical Analysis

Statistical analyses were performed using GraphPad Prism 8 (San Diego, CA, USA). Data are presented as mean ± standard error of the mean (SEM). Daily DAI scores and Grimace scores were analysed using a two-way analysis of variance (ANOVA) with Dunnett’s multiple comparisons test. All other data were analysed using one-way ANOVA followed by Dunnett’s post hoc test. Statistical significance is indicated as follows: “#” represents significance between healthy controls and vehicle-treated colitis, while “*” denotes significance between drug treatments and vehicle-treated colitis. A *p*-value of less than 0.05 was considered statistically significant.

## 5. Conclusions

This study provides compelling evidence that sub-therapeutic doses of THC combined with ZCZ011 or CBD offer a safe and effective strategy for managing both the inflammatory and metabolic components of IBD. Notably, the normalisation of GLP-1 and ammonia levels underscores the dual benefits of these treatments in alleviating colitis while addressing associated metabolic dysregulation and extraintestinal complications. This dual-action approach addresses key limitations of current therapies and emphasizes ECS modulation as a promising avenue for IBD treatment. By interlinking findings across acute and chronic colitis models and aligning them with emerging literature, this study emphasizes the significance of targeting both acute inflammation and chronic disease progression. Future studies should focus on elucidating the precise molecular mechanisms, particularly the downstream GLP-1 signalling pathways, cytokine suppression and glucose metabolism. Expanding these investigations to include other metabolic markers, such as lipid profiles and insulin sensitivity, will further advance the translational potential of cannabinoid-based therapies for IBD and related metabolic disorders.

## Figures and Tables

**Table 1:** Effect of treatments on blood haematology in DSS-induced acute colitis following 11 days of daily intraperitoneal administration.

Haematology	Healthy Control	Vehicle	2.5 mg/kg THC	5 mg/kg THC	10 mg/kg THC	20 mg/kg ZCZ011	30 mg/kg ZCZ011	40 mg/kg ZCZ011	20 mg/kg ZCZ011 + 2.5 mg/kg THC
WBC (10^9^/L)	6.8 ± 0.9	5.5 ± 0.5	7.5 ± 0.7	7.4 ± 0.6	4.2 ± 0.5	3.9 ± 1.0	4.1 ± 0.9	7.3 ± 0.7	7.0 ± 0.7
Lymphocytes (10^9^/L)	3.5 ± 1.2	3.8 ± 0.3	5.3 ± 0.6	5.1 ± 0.2	2.5 ± 0.6	3.2 ± 0.8	3.1 ± 0.6	4.9 ± 0.8	4.3 ± 1.1
Monocytes (10^9^/L)	0.2 ± 0.1	0.3± 0.02	0.34 ± 0.02	0.34 ± 0.1	0.18 ± 0.04	0.25 ± 0.1	0.2 ± 0.01	0.42 ± 0.2	0.26 ± 0.1
Granulocytes (10^9^/L)	3.1 ± 0.9	1.4 ± 0.1	1.9 ± 0.2	1.9 ± 0.4	1.5 ± 0.5	0.6 ± 0.2	0.9 ± 0.3	2.1 ± 0.4	2.5 ± 0.8
RBC (10^12^/L)	9.2 ± 0.1	9.5 ± 0.1	10 ± 0.1	10.1 ± 0.1	9.7 ± 0.3	8.9 ± 0.2	9.0 ± 0.2	9.8 ± 0.3	10.1 ± 0.1
Haemoglobin (g/L)	135.4 ± 2.1	143.0 ± 1.8	150.4 ± 1.8	151.4 ± 1.8	141.6 ± 4.6	145.5 ± 2.6	144.0 ± 4.2	152.8 ± 5.8	149.8 ± 1.8
Haematocrit (%)	42.0 ± 0.5	43.4 ± 0.8	45.3 ± 0.6	46.4 ± 0.4	44.1 ± 1.3	49.1 ± 1.3	47.2 ± 1.6	48.7 ± 1.3	47.0 ± 0.5
Platelets (10^9^/L)	1006.7 ± 56.1	893.1 ± 102.3	915.8 ± 65.7	969.6 ± 62.5	955.6 ± 139.3	812.3 ± 56.2	901 ± 38.1	981 ± 49.6	1354.6 ± 148.3 **
Mean platelet volume (fL)	4.9 ± 0.02	5.3 ± 0.2	5.4 ± 0.1	5.1 ± 0.1	5.0 ± 0.1	5.7 ± 0.8	5.9 ± 0.5	5.8 ± 0.1	5.0 ± 0.1
Platelet distribution width	16.3 ± 0.1	16.7 ± 0.3	17.1 ± 0.3	16.5 ± 0.1	16.6 ± 0.2	17.1 ± 0.3	17.3 ± 0.1	17.3 ± 0.3	16.4 ± 0.1
Procalcitonin (%)	0.5 ± 0.01	0.4 ± 0.1	0.5 ± 0.03	0.5 ± 0.1	0.46 ± 0.07	0.4 ± 0.03	0.5 ± 0.02	0.5 ± 0.02	0.60 ± 0.03 **

Data are presented as mean ± SEM; statistically significant differences were calculated using one-way ANOVA followed by Dunnet’s multiple comparisons test and are indicated by ** *p* < 0.01 compared to vehicle.

**Table 2:** Effect of the chronic treatments on haematology in DSS-induced chronic colitis.

Haematology	Healthy Control	Vehicle	20 mg/kg ZCZ011 + 2.5 mg/kg THC	20 mg/kg ZCZ011 + 10 mg/kg CBD
WBC (10^9^/L)	6.9 ± 1.2	5.0 ± 0.4	5.6 ± 0.8	5.5 ± 0.5
Lymphocytes (10^9^/L)	3.3 ± 1.4	2.8 ± 0.7	3.5 ± 0.8	4.1 ± 0.4
Monocytes (10^9^/L)	0.3 ± 0.1	0.2± 0.06	0.2± 0.04	0.2 ± 0.02
Granulocytes (10^9^/L)	3.3 ± 0.1	2.1 ± 0.5	1.7 ± 0.3	1.1 ± 0.2
RBC (10^12^/L)	9.4 ± 0.1	9.0 ± 0.2	9.4 ± 0.1	9.4 ± 0.2
Haemoglobin (g/L)	137.3 ± 2.0	134.1 ± 2.4	140.7 ± 1.7	141.8 ± 3.1
Haematocrit (%)	42.9 ± 0.5	41.4 ± 0.8	42.9 ± 0.4	42.9 ± 0.9
Platelets (10^9^/L)	1057 ± 56.0	986.3 ± 105.2	1066.3 ± 98.2	1140.5 ± 187.4
Mean platelet volume (fL)	4.8 ± 0.03	5.2 ± 0.1	4.7 ± 0.02	5.0 ± 0.1
Platelet distribution width	16.3 ± 0.06	16.9 ± 0.2	16.1± 0.04	16.5 ± 0.2
Procalcitonin (%)	0.5 ± 0.03	0.4 ± 0.02	0.4 ± 0.01	0.4 ± 0.01

Data are presented as mean ± SEM. No statistical differences among the groups as calculated using one-way ANOVA followed by Dunnet’s multiple comparisons test.

**Table 3:** Assessment of DAI score.

Clinical Markers of Colitis	Scores			
	0	1	2	3
Body weight loss	<5%	5–10%	11–15%	16–20%
Diarrhoea/stool consistency	Normal	Mild–soft, but still formed	Very soft/sticky	Watery diarrhoea/loose
Faecal blood score/rectal bleeding	Normal colour stool/no rectal bleeding	Positive hemoccult—slight (brown colour)/slight blood spotting in the anus	Positive hemoccult—darker (reddish)/significant presence of blood in the anus	Visible trace of blood/rectal bleeding

DAI score is calculated as the sum of the body weight loss score, stool consistency score and faecal blood/rectal bleeding score.

**Table 4:** Assessment of the Grimace score.

Pain Features/Scores	Scores		
	0	1	2
Orbital tightening	Not present	Closing of eyelid, narrowing of orbital area	Complete closure of eye with tightened orbital
Nose bulge	Not present	Slight bulging on the bridge of nose	Completely bulged nose
Cheek Bulge	Not present	Slight bulging of the cheek	Completely bulged
Ear position	Normal position	Ears moving towards the back	Folded ear forming a pointed shape
Whisker change	Normal whisker position	Whiskers pulled back/front	Clumping of whiskers
Movement/gait	Normal activity	Moves slowly	Moves only when provoked
Body position/hunching	Normal position	Slight tuck to abdomen	Fully hunched

The grimace score is calculated as the mean of all seven parameters outlined in the first column of the table.

## Data Availability

The original contributions presented in this study are included within the article. Additional data will be made available upon request and any further inquiries should be directed to the corresponding authors.
